# Heart Failure: Gaps in Knowledge and Failures in Treatment

**DOI:** 10.1371/journal.pmed.1001702

**Published:** 2014-08-12

**Authors:** Druin Burch

**Affiliations:** Public Library of Science, Cambridge, United Kingdom

## Abstract

*Please see later in the article for the Editors' Summary*

**Linked Research Article**

This Perspective discusses the following new study published in *PLOS Medicine*:

Callender T, Woodward M, Roth G, Farzadfar F, Lemarie J-C, et al. (2014) Heart Failure Care in Low- and Middle-Income Countries: A Systematic Review and Meta-Analysis. PLoS Med 11(8): e1001699. doi:10.1371/journal.pmed.1001699

In a systematic review and meta-analysis, Kazem Rahimi and colleagues examine the burden of heart failure in low- and middle-income countries.

In this week's *PLOS Medicine*, Rahimi and colleagues [1] report on the treatment of heart failure in low- and middle-income countries (LMICs). Based on 53 separate studies/datasets, they note that only 57% of patients are treated with angiotensin-converting enzyme inhibitors (ACEIs), 34% with beta-blockers, and 32% with mineralocorticoid receptor antagonists (with 95% confidence intervals, respectively, of 49%–64%, 28%–41%, and 25%–39%). The class of drugs used most often was that without proven prognostic benefit in heart failure, diuretics, which were provided for 69% (60%–78%) of patients. The authors conclude that “the use of evidence-based medications tends to be suboptimal”.

That is a euphemism, albeit a standard one. Outside the language of academic research, it could be bluntly rephrased as: “routinely doctors and healthcare systems cause needless death and major illness through failing to provide the care they should”. Recent work has suggested that 85% of all medical research is wasted through asking the wrong questions or asking questions badly, and more through difficulties in open access to useful knowledge [2]. Still more, though, is wasted when valuable and widely disseminated research results are not acted on. To notice, measure, and agonise over such waste is essential. So, however, is understanding the magnitude of its impact.

Geographical discrepancies in the diagnosis and treatment of heart failure [3] suggest that clinical opinions and behaviour vary across the world. One speculation is that clinical diagnoses of heart failure are often plain wrong [4]. Another concern is the difference between heart failure with impaired or preserved left ventricular systolic function. The two types split fairly evenly among those patients deemed clinically to have overt congestive heart failure [5],[6] yet may not do so in other populations. In one echocardiographic study of unselected community participants, 6% of those aged over 45 years had moderate or severe diastolic failure compared to 2% with systolic failure [7]. Community-based studies from high-income countries show asymptomatic systolic [8] and diastolic failure are common and harmful. Estimates of the burden of disease will be swayed by how they are sought and by the availability of diagnostic tests such as echocardiography and brain natriuretic peptide measurements. Methods for identifying heart failure in the studies reviewed by Rahimi and colleagues were strikingly inconsistent, as the criteria or guidelines used varied [1]. In one study diagnosis rested entirely on echocardiography, while in 26 it was solely the judgement of the physician.

Diastolic failure is more likely in the elderly [9], is very much more likely in women (who are under-represented in trials partly as a result) [10], is less associated with previous heart attack and more with hypertension [11], and comes without any neat technologically derived indicator of apparent severity. Diastolic failure has a prognosis that, despite being somewhat better than that of systolic failure, remains poor [12], and it confers similar reductions in quality of life [13]. It is resistant to treatment, with no specific therapies having been shown to improve outcomes [14]. Rahimi and colleagues were unable to determine what proportion of those identified with heart failure had systolic impairment. “Few studies reported the LVEF [left ventricular ejection fraction] of patients, and fewer still separated data by LVEF”, they report, adding that “consequently, it is not possible to make strong conclusions about the adherence of practice to evidence-based practices worldwide”. They note that “data from the EuroHeart Failure Survey II of 30 high-income European countries also demonstrated poor medical management” [1]. Compared with Rahimi and colleagues' finding of 57% of heart failure patients in LMICs getting ACEIs, 34% beta-blockers, and 32% mineralocorticoid receptor antagonists, the EuroHeart Failure Survey II found figures of 71%, 61%, and 48%, respectively. It reported 34% of patients hospitalised with acute heart failure to have preserved left ventricular ejection fractions [15] but did not look at medications relative to left ventricular ejection fraction. Without knowing whether drugs are given to those with systolic impairment, it is impossible to calculate harms done by omission or to speculate on the costs of prescribing agents to those with diastolic failure shown not to benefit from them.

The finding that nearly half of heart failure in Africa is caused by hypertension suggests a substantial burden of potentially preventable disease and highlights uncertainty over the balance of systolic and diastolic failure. That only one population-based study estimating the incidence or prevalence of heart failure in LMICs could be found is remarkable; directing scarce resources needs better information. In South Africa, 16% of deaths in 2011 were attributed to “diseases of the circulatory system”, up from 14% in 2009 [16]. The proportion of deaths that can be attributed to heart failure is unclear. In places where death certification is less reliable, verbal autopsies have estimated cardiovascular disease to be responsible for 8%–14% of deaths in sub-Saharan Africa and 18%–22% of deaths in Bangladesh [17], but again the proportion of these due to systolic heart failure is opaque. Establishing the burden of systolic and diastolic heart failure is required to put estimates of under-treatment into perspective. Trial data from high-income countries suggests substantial benefits of treatment for those with reduced ejection fractions. ACEIs are associated with a 15%–27% reduction in death [18], spironolactone [19] with a 30% reduction, and beta-blockers with the same (although the number of events in beta-blocker trials is surprisingly small, and the mortality benefit therefore “only moderately robust”) [20],[21]. Assuming these effects are additive, even a conservative estimate would suggest the difference between evidence-based treatment and its absence to be of the order of a halving in death rates attributed to systolic heart failure. If this is what increased treatment rates would accomplish in LMICs, effort could then be focused on identifying barriers to treatment and how to overcome them, while approaches to diastolic dysfunction might focus on preventative treatment of hypertension and palliative treatment of symptoms.

Heart failure is a growing global problem. Variations in diagnostic strategy undermine establishment of an optimal response, as does the focus in clinical trials on atypically youthful, male populations with systolic dysfunction. Future progress in reducing the burden of heart failure depends on attending to what makes the most difference, not what makes for the easiest trials. Treatment matters, even when treatment is palliative, but reducing cardiovascular risk in an effort to prevent heart failure may be the most important strategy. Determining what works best is important not only in the resource-poor settings of LMICs. Obesity and exercise are modifiable risk factors for the development of heart failure, but our ability to affect them is limited compared to our power to alter blood pressure and lipids.

Rahimi and colleagues highlight likely therapeutic failures when it comes to prolonging and improving the lives of those suffering heart failure in LMICs [1]. The consequences of these failures, and the costs and potential of improving them, need to be better established. Hard choices about whether to direct resources to treating the causes or the manifestations of heart failure may evaporate in the light of greater knowledge, since many of the interventions for one may aid the other. What works—to what extent and for patients identified by what method and in which setting—remains to be properly shown. The gap between suboptimal treatment of heart failure and what is achievable represents not just a failure to practice evidence-based medicine but a greater gap in knowledge and the research agenda.

## Abbreviations

ACEI: angiotensin-converting enzyme inhibitor
LMICs: low- and middle-income countries
